# Analysis of the factors motivating HCV-infected patients to accept interferon therapy

**DOI:** 10.1186/1756-0500-5-470

**Published:** 2012-08-29

**Authors:** Yumiko Nagao, Michio Sata

**Affiliations:** 1 Department of Digestive Disease Information & Research, Kurume University School of Medicine, 67 Asahi-machi, Kurume, Fukuoka, 830-0011, Japan; 2 Division of Gastroenterology, Department of Medicine, Kurume University School of Medicine, Kurume, Fukuoka, 830-0011, Japan

**Keywords:** Hepatitis C virus, Interferon therapy, Chronic hepatitis C, Hepatocellular carcinoma, Oral lichen planus

## Abstract

**Background:**

The aims of this study were to analyze factors motivating the acceptance of interferon (IFN) therapy and to clarify the prevalence of oral mucosal diseases in hepatitis C virus (HCV)-infected Japanese patients treated with IFN.

**Findings:**

A total of 94 HCV-infected patients who were admitted to our hospital for IFN therapy were asked questions regarding their motivation to accept IFN therapy and were investigated for the presence of oral lichen planus (OLP) before and during IFN treatment. Recommendation and encouragement from other people were the most common factors motivating the acceptance of IFN therapy (49/94, 52.13%). The other motivators were independent decision (30.85%), economic reasons (5.32%), and others. According to multivariate analysis, three factors – sex (male), retreatment after previous IFN therapy, and independent decision to accept IFN therapy - were associated with patients after curative treatment of hepatocellular carcinoma (HCC). The adjusted odds ratios for these three factors were 26.06, 14.17, and 8.72, respectively. The most common oral mucosal lesions included OLP in 11 cases (11.70%). One patient with OLP had postoperative squamous cell carcinoma of the tongue. The rate of sustained virological response (SVR) was 45.45% in cases with OLP and 54.55% in cases without OLP. There were no patients who discontinued IFN therapy because of side effects such as oral mucosal diseases.

**Conclusions:**

We should give full explanation and recommend a course of treatment for a patient to accept IFN therapy. The system to support liver disease as well as oral diseases is also necessary for patient treated for IFN therapy.

## Findings

### Background

Japanese hepatitis C virus (HCV)-infected patients tend to be older than those in other countries and their older age favors the onset of hepatocellular carcinoma (HCC), leading to an increased mortality rate [1,2]. The number of deaths from HCC continues to rise in Japan, where about 80% of HCC are caused by HCV and 10% by hepatitis B virus (HBV) [1,2].

Interferon (IFN) therapy for chronic hepatitis C is the only treatment that enables complete elimination of the virus. In recent years, pegylated IFN (Peg-IFN) and ribavirin (RBV) combination therapy has been the standard treatment for chronic hepatitis C. It has been shown that IFN therapy contributes to the prevention of occurrence of HCC and to improvement in long-term prognosis [3-6]. In view of the current circumstances, Japan’s Ministry of Health, Labor and Welfare introduced in April 2008 a support system for medical costs for HCV and HBV carriers receiving IFN therapy [7].

We reported previously factors interfering with the acceptance of IFN therapy by HCV-infected patients at eight facilities (clinics/hospitals) [8,9]. Why is IFN therapy not used more widely for treatment of HCV carriers in Japan? Multivariate analysis demonstrated that treatment facilities, sex and the presence or absence of complications were factors associated with the risk that patients would decline IFN therapy. Female patients were more likely than male patients to decline IFN therapy because of worries about the adverse effects of the therapy.

The sustained virological response (SVR) rate after 48 weeks of Peg-IFN/RBV therapy at the standard dose is approximately 40 to 50%, while the frequency of adverse events in combination therapy with Peg-IFN/RBV is relatively high (20-64%) [10-12]. Among the side effects of IFN therapy, oral lesions might be easy to be overlooked and, among the side effects in a Japanese Phase III trial of Peg-IFN/RBV, oral mucosal disease and dental problems have been documented in patients with chronic hepatitis C.

HCV infection is associated with several clinical and biological extrahepatic manifestations [13]. These include hematologic diseases such as cryoglobulinemia and lymphoma, renal diseases such as membranoproliferative glomerulonephritis, autoimmune disorders such as thyroiditis, and dermatologic conditions such as lichen planus (LP) and porphyria cutanea tarda.

In the present study, we analyzed the factors motivating the acceptance of IFN therapy and investigated the development of oral mucosal disease at the time of IFN therapy of HCV-infected patients.

### Methods

#### Patients

A total of 94 HCV-infected patients who were admitted to the Kurume University Hospital from October 2009 to September 2011 for treatment with IFN monotherapy, Peg-IFN monotherapy or Peg-IFN/RBV combination therapy were studied (Table 1). All patients were admitted in order to achieve sustained eradication of HCV. Seventy-two patients had not received previous interferon therapy and 22 had relapsed after previous IFN therapy. The 94 patients were 58 men and 36 women with a mean age of 57.01 ± 11.55 years. There were 9 patients (8 men and one woman) with histories of curative treatment for HCV-related HCC. They had been monitored carefully for relapse over a year or more. Exclusion criteria of this study were patients with long-term maintenance IFN therapy and participants in a clinical trial.

**Table 1:** Characteristics of the 94 patients

**Characteristics**		**n**	**%**
Sex	male / female	36/58	
Age	(mean ± SD), years	57.01 ± 11.55	
Age groups	20-29 years	3	3.19%
	30-39 years	5	5.32%
	40-49 years	15	15.96%
	50-59 years	22	23.40%
	60-69 years	38	40.43%
	70-79 years	11	11.70%
Employment	unemployed	42	44.68%
	employed	52	55.32%
Diagnosis of liver disease	AH-C	1	1.06%
	CH-C (after HCC treatment)	87 (5)	92.55%
	LC-C (after HCC treatment)	6 (4)	6.38%
IFN therapy	IFN beta monotherapy	1	1.06%
	IFN beta induction therapy followed Peg-IFN alpha 2b/RBV	12	12.77%
	Peg-IFN alpha 2b/RBV	60	63.83%
	Peg-IFN alpha 2a/RBV	11	11.70%
	Peg-IFN alpha 2a monotherapy	7	7.45%
	Peg-IFN alpha 2b/RBV → Peg-IFN alpha 2a monotherapy	2	2.13%
	Peg-IFN alpha 2b/RBV → Peg-IFN alpha 2a monotherapy → Peg-IFN alpha 2a/RBV	1	1.06%

SD, standard deviation; AH-C, acute hepatitis C; CH-C, chronic hepatitis C; LC-C, liver cirrhosis type C; HCC, hepatocellular carcinoma; IFN, interferon; Peg-IFN, pegylated interfeon; RBV, ribavirin.

According to the clinical pathway, all patients had checkups for oral mucosal diseases in the Digestive Diseases Center at our Hospital. In this Digestive Diseases Center, physicians, surgeons, radiologists and an oral surgeon examine each patient in their own specialized area. Each patient was advised by an oral surgeon about the presence of oral infection before commencing IFN treatment. All patients who received IFN therapy for chronic HCV liver disease at our hospital were required to undergo hospitalization for two weeks for therapeutic management and education about liver diseases. After the patients left the hospital, those who felt an uncomfortable feeling in the mouth such as reticular oral lichen planus (OLP) and xerostomia were free to consult the same oral surgeon. The study complied with the ethical guidelines of the Declaration of Helsinki. Informed consent was obtained from all patients after the purpose and methods of the study were explained.

#### Factors motivating HCV-infected patients to accept IFN therapy

All patients answered the oral surgeon’s questions about their motivation for accepting IFN therapy. If a physician who treats directly chronic liver disease questions a patient, bias error occurs to patient’s answer. Therefore oral surgeon, member of a healthcare team interviewed to keep neutrality and equality. Specific questions based on validation reports interfering with the acceptance of IFN therapy by HCV-infected patients [8,9], including 1) recommendation and encouragement from other people, 2) independent decision, 3) economic reasons, 4) outcome of same diseases in close associates, 5) increased leisure time, 6) gathering medical information, 7) recovering from an extrahepatic disease, and 8) the others, are listed in Table 2. 

**Table 2:** Factors motivating the acceptance of IFN therapy

	**Question items**	**Multiple answers**		**Only answer**		**Only answer**	
		**n**	**%**	**n**	**%**	**n**	**%**
Recommendation and encouragement from other people	Recommendation from a primary care doctor	62	65.96%	18	19.15%	49	52.13%
	Recommendation from a hepatologist	71	75.53%	18	19.15%		
	With family’s encouragement	40	42.55%	8	8.51%		
	With the encouragemant of friends and acquaintances	15	15.96%	4	4.26%		
	With encouragement from a government office and healthcare center	2	2.13%	0	0.00%		
	With encouragement from a patient advocacy group for liver disease	1	1.06%	1	1.06%		
Independent decision	Overcoming an illness by patient’s own determination	79	84.04%	20	21.28%	29	30.85%
	Hope of longevity for the pateint and his family	66	70.21%	9	9.57%		
Economic reasons	Reducing hepatitis virus-infected patient’s cost of medical care in the Japanese medical system from the beginning of 2008.	51	54.26%	4	4.26%	5	5.32%
	Coping with lower income before the official retirement age	1	1.06%	1	1.06%		
Outcome of same diseases from the people nearest	Awareness of people such as family and friends who have recovered from illness	9	9.57%	2	2.13%	5	5.32%
	Awareness of death in the family and friends having the same liver diseases	12	12.77%	3	3.19%		
Increased leisure time	Devotion of certain amounts of time to the treatment of liver disease by reason of retirement and corporate downsizing	12	12.77%	2	2.13%	3	3.19%
	Devotion of certain amounts of time to the treatment of liver disease free of elders who need care and student taking an entrance exam	25	26.60%	0	0.00%		
	Coordination of work schedules to receive the treatment	34	36.17%	1	1.06%		
Gathering medical information	Participation in seminar for liver disease with patients	21	22.34%	0	0.00%	1	1.06%
	Reading a newspaper, book or pamphlet	41	43.62%	0	0.00%		
	Watching a TV program	28	29.79%	1	1.06%		
	Gather information from the internet	21	22.34%	0	0.00%		
	Watching TV reports of lawsuits of hepatitis	23	24.47%	0	0.00%		
Recovering from an extrahepatic disease	Concentratation on the treatment of liver diseases after recovering from an extrahepatic disease	7	7.45%	1	1.06%	1	1.06%
The others	Dislike receiving injections for liver supporting therapy thrice weekly for many months	4	4.26%	1	1.06%	1	1.06%

#### Examination of oral mucosal disease

We used the headband fiber (50-100-10, Daiichi Medical Co., Ltd.) with a brightness of 34,000 luces for mucosal examination. Oral biopsy was performed on some patients. The diagnosis of OLP was made on the basis of clinical and histopathological features. Salivary flow was measured in patients with dry mouths. We used a simple and low-cost test for detection of xerostomia and this required chewing on a piece of gauze for 2 min. A salivary flow rate of below 2 g/2 min was judged as decreased salivary secretion.

#### Statistical analysis

All data are expressed as mean ± standard error. Differences between the two groups were analyzed using Wilcoxon’s test. Differences were judged significant for p <0.05 (two-tailed). Adjusted odds ratios were calculated using logistic regression analysis. All statistical analyses were conducted using JMP Version 6 (SAS Institute, Cary, NC, USA). The level of statistical significance was defined as 0.05.

### Results

#### Factors motivating why patients accepted IFN therapy

Table 2 lists multiple answers and the principal factors motivating patients to accept IFN therapy. Of 94 patients who received IFN therapy, recommendation to them by a physician**,** such as a primary care doctor or hepatologist, family or a friend was the most common motivation for choosing the therapy (49 cases, 52.13%). The other motivators were: independent decision (29/94, 30.85%), economic reasons (5/94, 5.32%), outcome of the same liver diseases in close associates (5/94, 5.32%), increased leisure time (3/94, 3.19%), gathering medical information (1/94, 1.06%), and recovering from an extrahepatic disease (1/94, 1.06%).

The principal reasons for nine patients who had previous HCC treatment accepting IFN therapy were as follows: overcoming an illness by the patient’s own determination (4 cases), hope of longevity for the patient and his family (2 cases), recommendation from a hepatologist (2 cases), and recommendation from a primary care doctor (1 case).

#### Prevalence of OLP among patients with HCV infection who received IFN

The prevalence of OLP was 11.7% (11/94). The average age of those with OLP (62.18 ± 6.43 years), 2 men and 9 women, was greater than that of 83 non-OLP patients (56.33 ± 11.92 years) but there was no significant difference. The distribution of OLP according to site of occurrence was: buccal mucosa 11 (100%), tongue 3 (27.27%), lower lip 2 (18.18%), gingiva 1 (9.09%), oral floor 1 (9.09%), and soft palate 1 (9.09%), among 19 sites in 11 patients (Table 3). The erosive or reticular type of OLP was found in 7 (63.63%) and 4 (36.36%) patients, respectively. Onset of OLP was: before IFN therapy (8, 72.72%) and during IFN therapy (3, 27.27%). Symptoms of three OLP patients with erosive type worsened during IFN treatment, but those reduced their symptoms by application of steroid. We did not need to reduce dose of IFN and/or RBV. The effect of IFN therapy in 11 OLP with patients were: SVR (5/11, 45.45%) and non-SVR (6/11, 54.55%).

**Table 3:** Distribution of oral mucosal diseases with HCV-related liver diseases

**No**	**Sex**	**Age**	**Liver disease**	**Oral lichen planus**								**Oral cancer**	**Oral leukoplakia**	**Aphthous stomatitis**	**Xerostomia**	**Oral candidosis**	**Traumatic ulcer**	**Benign tumor**
				**(n = 11)**								**(n = 1)**	**(n = 1)**	**(n = 3)**	**(n = 3)**	**(n = 2)**	**(n = 1)**	**(n = 3)**
				**buccal mucosa**	**gingiva**	**tongue**	**lower lip**	**oral floor**	**soft palate**	**type**	**onset**	**tongue**	**tongue**				**tongue**	
1	F	55	CH-C	◯		◯				erosive	during IFN therapy							
2	F	58	CH-C											◯				
3	M	54	CH-C	◯	◯	◯	◯			erosive	before IFN therapy							
4	M	62	CH-C	◯					◯	reticular	during IFN therapy							
5	F	66	CH-C	◯						erosive	before IFN therapy							
6	M	61	CH-C															◯
7	F	55	CH-C	◯						reticular	during IFN therapy							
8	F	62	CH-C												◯ (with fissured tongue)			
9	F	72	CH-C	◯						erosive	before IFN therapy							
10	F	70	CH-C												◯ (with fissured tongue)			
11	M	58	CH-C															◯
12	F	57	CH-C	◯		◯		◯		erosive	before IFN therapy	◯ (post ope.)						
13	F	60	CH-C											◯				
14	F	71	LC-C	◯						erosive	before IFN therapy				◯			
15	F	66	CH-C													◯		
16	M	67	CH-C											◯				
17	F	66	CH-C										◯					
18	F	64	CH-C	◯			◯			erosive	before IFN therapy							
19	F	61	CH-C	◯						reticular	before IFN therapy							
20	F	29	CH-C														◯	
21	F	67	CH-C	◯						reticular	before IFN therapy							
22	F	49	CH-C													◯		
23	M	48	CH-C															◯

CH-C: chronic hepatitis C, LC-C: HCV-related liver cirrhosis, IFN: interferon therapy, ope: operation.

The circles indicate the presence of the disease.

The other oral diseases were: postoperative squamous cell carcinoma of the tongue (1/94, 1.06%), leukoplakia of the tongue (1/94, 1.06%), aphthous stomatitis (3/94, 3.19%), xerostomia (3/94, 3.19%), oral candidosis (2/94, 2.12%), traumatic ulcer of the tongue (1/94, 1.06%), and oral benign tumor (3/94, 3.19%). Two patients (2.12%) had fissured tongue. A 57-year-old woman with OLP had a history of resection of squamous cell carcinoma of the tongue (T1N0M0, stage I) nine years previously. Three patients with xerostomia had salivary flows below the normal value (the average flow, 1.09 ± 0.07 g/2 min).

There were no patients who discontinued IFN therapy because of side effects such as OLP and the other oral diseases. We commenced the usual therapeutic doses of IFN regardless of the presence of oral mucosal lesions.

#### Multivariate analysis of the patients with HCC after curative treatment

According to multivariate analysis, three factors, sex (male), retreatment after previous IFN therapy, and independent decision to accept IFN therapy, were characteristic factors of the patients after curative treatment of HCC. The adjusted odds ratios for these three factors were 26.06, 14.17, and 8.72, respectively, and each was statistically significant (Table 4).

**Table 4:** Multivariate analysis of the patients with HCC after curative treatment

	**Adjusted odds ratio (95% confidence interval)**		**P value**
Sex (male)	26.06	(3.44 - 605.65)	0.0078
Retreatment after previous IFN therapy	14.17	(2.38 - 127.99)	0.0068
Independent decision to accept IFN therapy	8.72	(1.47 - 77.00)	0.0257

#### Discussion

Japan has operated a universal health insurance system since 1961. Any person who has an address in Japan must enroll for public health insurance. Based on the insurance benefits, the medical fees that an individual pays upon receiving a medical examination at a hospital, dental clinic, etc. are reduced.

It is believed that between one and two million Japanese people are chronically infected with HCV [1,2]. In Japan, the Ministry of Health, Labor and Welfare alleviated the economic burden of therapy for diseases associated with HCV and HBV infection in April 2008 and expanded this system for the treatment of HBV carriers with nucleoside analogues in April 2010 [7]. The program is seen as one way of reducing the cost of medical care in the Japanese medical system for patients infected with hepatitis B and C viruses. Medical care certificates for receipt of IFN therapy were issued to 44,731 people from April 2008 to May 2009. The certificates were issued to 26,594 people from April 2009 to May 2010. The certificates to receive IFN therapy and to receive nucleoside analogues therapy were issued to 28,797 and 38,038 people, respectively, from April 2010 to May 2011.

In this study, I believe we were able to collect unbiased answers from the subjects because the oral surgeon who questioned the patients was not involved directly with IFN treatment. Most of the motivators for patients to receive IFN therapy were recommendation and encouragement from a physician such as primary care doctor, rather than economic reasons. Firstly patients sought to gather information about liver disease from newspapers, the internet, etc. Subsequently, patients finally accepted, IFN therapy after they were satisfied with the explanations from their doctor. The patients who had received curative treatment for HCC decided for themselves to accept IFN therapy. What should be done about the medical approach for patients who accept IFN therapy?

Medical informed consent is essential for the physician’s ability to diagnose and treat patients**,** as well as the patient’s right to accept or reject clinical evaluation, treatment, or both [14]. Patients need to participate in the informed consent process to understand the risk-benefit relationship of the proposed treatment strategy.

Previously, we analyzed the factors preventing the implementation of IFN therapy in terms of the one to one relationship between the patient and primary care physician in X town (adult population: 7,389), in northern Kyushu, Japan, where the prevalence of HCV infection is the highest in the country [8,9]. Of 139 patients to whom attending physicians recommended IFN therapy, 92 (66.2%) agreed to receive the treatment. In contrast, 74 (86.0%) of 86 hospital patients (treated by liver specialists) agreed to receive IFN therapy but only 18 (34.0%) of 53 clinic patients (treated by non liver-specialists) did so. The difference was attributable to the intensity of the effort and the strength of the explanations or recommendations given by the physicians to the patients. In logistic regression analysis, the adjusted odds ratios on treatment facilities, sex and complications were 18.06, 3.65, and 3.63 respectively, indicating that there were significant differences. It is also essential to devise measures to create cooperation between hospitals and clinics, and to improve communication between physicians and their patients.

HCV affects other organs, as well as the liver. Extrahepatic manifestations can be found in the mucosa, skin, eyes, salivary gland, joints, kidneys, and immune system. Cacoub *et al*. reported that 38% of patients with HCV infection manifested at least one extrahepatic manifestation [15]. We reported that the prevalence of mucous or cutaneous LP, type 2 diabetes mellitus, hypertension, thyroid dysfunction, and extrahepatic malignant tumor were 19.5%, 21.8%, 28.7%, 20.7%, and 9.2%, respectively [16]. LP is associated frequently with HCV infection and can be exacerbated by IFN therapy. As regards the effects of IFN therapy on LP lesions, there is a report of their improvement [17], a report of LP manifestation triggered by IFN [18,19], and a report of aggravation of LP [20-23]. In long-term observation for 3 years or longer, we reported some OLP lesions (all reticular type) were improved**,** not only macroscopically but also in histopathologic examination [24]. The erosive type of OLP, in particular, can cause spontaneous pain during eating and tooth-brushing, while a patient is receiving IFN therapy for HCV infection.

Before a patient with HCV infection receives IFN treatment in our hospital, that patient must undergo diagnostic evaluation of the mouth, the eyes, the mental state and the circulatory disease. We reported that six of 570 patients could not commence IFN therapy despite their admission, because of dental problems such as periodontitis, pupitis, and pericoronitis [25]. Treatment of dental infections is required before the commencement of IFN therapy for HCV infection. A cooperative system involving the various medical specialists leads to acceptance of IFN treatment.

We have been holding regional seminars, so-called “seminar for digestive disease”, since 2005 to improve understanding of digestive diseases such as liver disease and extrahepatic manifestations by patients, their families, and healthcare workers. A total of 3,776 people have attended the lectures to date. It is also important that we provide up to date knowledge of liver disease to many people. We have to understand that various factors and motivations behind behavior, which lead to refusal or acceptance of IFN therapy, vary greatly between individual patients and need to support the patients and their families by a specialist for each area before, during, and after treatment.

### Conclusions

In conclusion, we analyzed factors motivating the acceptance of IFN therapy by HCV-infected patients and showed the importance of the effort and the strength of the explanations or recommendations given by physicians to patients. Physicians also should be aware of OLP occurrence during IFN treatment of patients with hepatitis C.

## Abbreviations

HCV: hepatitis C virus; HBV: hepatitis B virus; CH-C: chronic hepatitis C; LC-C: liver cirrhosis type C; HCC: hepatocellular carcinoma; OLP: oral lichen planus; IFN: interferon; Peg-IFN: pegylated interferon; RBV: ribavirin; SVR: sustained virological response.

## Competing interests

The authors declare that they have no competing interests.

## Authors’ contributions

YN carried out most of the data collection, designed the study, and drafted the manuscript. MS contributed to data analysis. Both authors read and approved the final manuscript.
